# Monolithic focus-tunable lens technology enabled by disk-type dielectric-elastomer actuators

**DOI:** 10.1038/s41598-020-73666-0

**Published:** 2020-10-09

**Authors:** Bong Je Park, Suntak Park, Meejeong Choi, Seung Koo Park, Sungryul Yun, Eunjin Shin, Jae Woong Yoon

**Affiliations:** 1 Artifical Intelligence Research Laboratory, Electronics and Telecommunications Research Institute (ETRI), Daejeon, 34129 Korea; 2 Department of Physics, Hanyang University, Seoul, 04763 Korea

**Keywords:** Applied optics, Imaging and sensing, Mechanical engineering

## Abstract

We propose a monolithic focus-tunable lens structure based on the dielectric-elastomer actuator (DEA) technology. In our focus-tunable lens, a soft lens and radial in-plane actuator mimicking the ocular focal-tuning mechanism are constructed in a single body of an optimized dielectric-elastomer film. We provide device fabrication methods including elastomer synthesis, structure formation, and packaging process steps. Performance test measurements show 93% focal tunability and 7 ms response time under static and dynamic electrical driving conditions, respectively. These performance characteristics are substantially enhanced from the previous polylithic DEA tunable lens by a factor 1.4 for the focal tunability and a factor 9.4 for the dynamic tuning-speed limit. Therefore, we obtain greatly enhanced focal tuning control in a remarkably simple and compact device structure.

## Introduction

Optical imaging systems have a wide variety of essential uses over fundamental sciences, defense industry, security surveillance, and consumer electronics as well. Proper formation of optical images for desired visual objects requires focus-tunable elements in general. Focus-tuning mechanisms that have been established so far include translational movement of lenses, refractive index change^1^, and surface curvature change^2^. Recently, the latter two approaches attract considerable attention because they enable compact, light-weight, and high-speed imaging systems favorable for emerging application areas such as robotics, drones, autonomous vehicles, medical instruments, and many others^3^.

The refractive-index change approach using polarization-dependent electro-optic effect in liquid crystals produces electrically driven focus-tunable lens units. This method can generate continuously variable focal length in a refractive power range between ± 2000 m^−1^ and is capable of high-speed operation with a dwell time around a few ms^4–7^. Although there are limitations associated with inherent polarization dependence and unavoidable electrode inclusion on the optical path, this approach has been very promising for adoptive or smart optical systems in various application areas in practice.

Alternatively, dielectric-elastomer tunable lenses and focusing mirrors have been developed as an electrically driven tunable-curvature lens technology^8–16^. In this approach, desired focal tuning is obtained by applying compressive or tensile force on a curved soft medium as ocular organs of vertebrates work. Therefore, technical issues associated with polarization dependence or electrode inclusion on the optical path are not necessarily involved in this approach. Importantly, the driving mechanisms using capacitor-type electrode pairs are considerably simpler than phase-type spatial light-modulator architectures necessarily included in the liquid–crystal-based index-change approaches. In addition, dielectric-elastomer tunable lenses are superior to oil-droplet lenses^17^ in physical robustness against inertial impact or gravitational deformation. Therefore, in spite of disadvantages related to high operation voltage and limitations in high-speed applications, further development for dielectric-elastomer tunable-curvature lenses is of great interest for their potential uses in highly compact or mechanically flexible imaging systems for artificial ocular components, endoscopic devices, and micro-robotic vision units.

Various lens structures based on electro-active polymer actuators have been proposed toward this end^8–16,18,19^. Liquid-filled elastomer membrane structures take advantages of high physical mobility of the lens-forming optical fluid to yield wide tuning ranges over 100% of an unforced focal length and a response time ~ 0.2 ms^8–12^. In another approach, all solid-state elastomer lenses integrated with carefully designed electrode patterns have been suggested to obtain better performance stability and mechanical robustness^13–16^. Previously, we have developed an electrically tunable lens architecture that is constructed by integrating a separately fabricated dielectric-elastomer actuator (DEA) and soft lens connected with sector-form vertical coupler mechanisms^13^. Therein, electrically-driven focal-length tunability of 65% and 67 ms response time are obtained using the DEA tunable-curvature lens concept. Although tunable-curvature elastomer lenses are presently under intensive development for their potential applications in numerous emerging industrial areas, there are still challenges in structural complexity, scalability, and subsequent performance restrictions.

In this paper, we propose an all-solid-state monolithic DEA tunable-curvature lens technology which takes advantages of remarkably enhanced structural simplicity, compactness, and performance characteristics as well. The proposed tunable lens consists of a single optimally-synthesized DEA membrane under strong prestretch condition to enable purely in-plane radial actuation of lens diameter and consequent curvature change. Driven by electrically-induced radial force on the soft lens in a single body, our proposed device provides a reasonably wide focal-tuning range of 93% of a minimum focal length in the active tuning range and quick response time ~ 7 ms. These performance characteristics are substantially improved from the previous polylithic all-solid-state DEA tunable-curvature lens^13^ as we expect in our proposed monolithic structure approach.

## Results

### Device structure and fabrication

**Figure 1:**
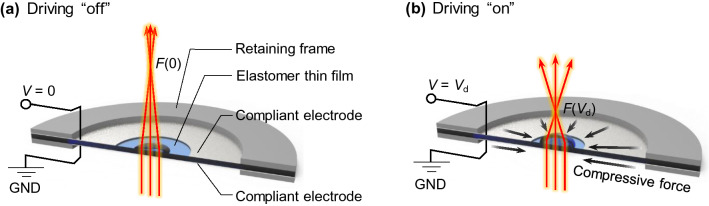
Structure and operation principle of a monolithic focus-tunable lens. (**a**) Lens structure and ray diagram for driving signal “off” (*V* = 0). (**b**) Ray diagram for driving signal “on” (*V* = *V*_d_). *F*(*V*) indicate driving-voltage-dependent focal length. In (**b**) for an “on” state, attractive electrostatic force between the top and bottom electrodes creates radially compressive force on the elastomer thin film, and the elastomer lens at the center shrinks to yield shorter focal length *F*(*V*_d_) than *F*(0) for the “off” state.

Structure geometry and operation principle of a monolithic DEA tunable-curvature lens in this study are illustrated in Fig. 1. A lens and a surrounding disk-type DEA are formed in a single pre-stretched elastomer film mounted between a pair of rigid retaining frames as shown in Fig. 1a. For a non-zero voltage applied between the two compliant electrodes, electrostatic attraction between the two annular electrodes produces radially compressive force directing towards the lens at the center, and subsequent modification of the lens surface curvature leads to a focus at a shorter distance as depicted in Fig. 1b.

Creating a monolithic tunable lens operating this way, it is crucial to have certain desired mechanical, electrical, and optical properties all together in a dielectric-elastomer film since an acceptably strong DEA and easily deformable transparent lens are to be constructed in a single body of an elastomer membrane. Hence, we synthesize an optically transparent custom dielectric elastomer which has dielectric constant as high as possible and Young's modulus as low as possible. Lower Young’s modulus produces larger elastic strain on the lens to be tuned while higher dielectric constant makes working voltage limit higher so that the actuator can exert stronger compressive power. In the optimal elastomer synthesis, we use a poly(dimethylsiloxane-*co*-methylvinyl-siloxane) and poly(dimethylsiloxane-*co*-methylsiloxane) compound at a mixing ratio 95:5 wt%. Initial modulus, maximum strain, and dielectric constant of this optimal elastomer film are 390 kPa, 310%, and 2.8 at 1 kHz, respectively, as described in our recent report^19^. Therein, we provide comprehensive information on polymer synthesis procedures and characterizations for chemical, mechanical, and optical properties in greater detail. Optical transmittance of this elastomer film for thickness 500 μm is higher than 93% over the entire visible spectrum from 400 to 700 nm as shown in Supplementary Fig. 1.

**Figure 2:**
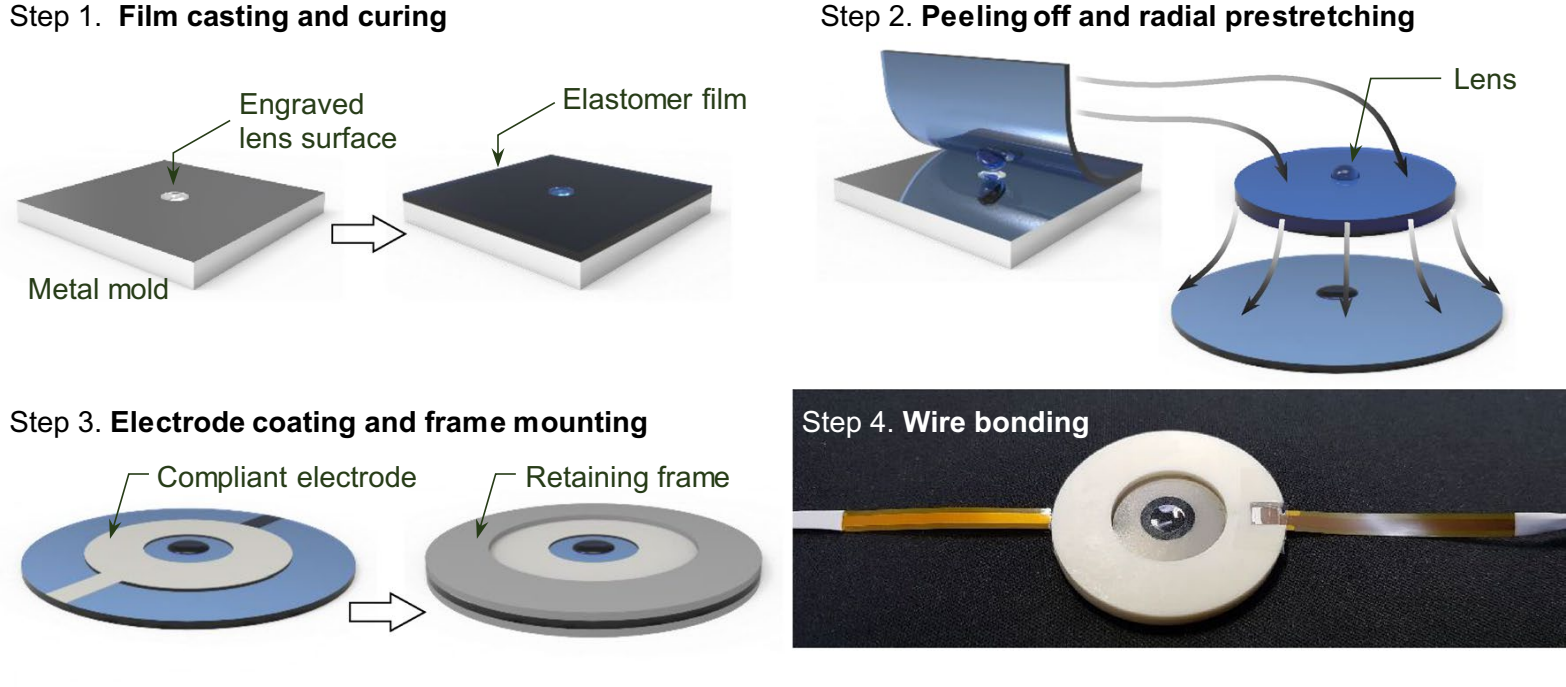
Fabrication process flow for a monolithic focus-tunable lens. Four process steps in sequential order are illustrated clockwise from a bare metal mold on top left to a photograph of a successfully fabricated device sample on bottom left. Footprint diameter of the final device including the retaining frames is 40 mm and the inner active region is 20-mm wide.

Once the optimal elastomer synthesis is established, we fabricate a monolithic tunable lens following a process flow illustrated in Fig. 2. The fabrication process starts with a metal mold (stainless steel) with engraved lens surface. Different size and radius of curvature of an engraved lens surface on a metal mold are carefully selected depending on the desired focal length and numerical aperture of the final polymer lens device due to subsequent process steps. The optimally synthesized dielectric elastomer in its liquid phase is casted on the metal mold with a bar coater and cured at 80 °C for 2 h. The casting condition are tuned to yield a cured-film thickness ~ 200 μm. The cured film is then peeled off from the metal mold. The film is stretched by a strain ~ 100%. This prestretch step is crucial for creating purely in-plane radial actuation of the film^8,9,13,18,20^ and consequent change in the curvature of the lens at the center. The optimal strain value ~ 100% is empirically obtained so that strain change under electrical driving condition becomes maximal, as similar value for an optimal in-plane radial DEA was reported in^13^. The annular compliant electrode pair is formed by spray-coating of silver nanowire suspension. The silver nanowire suspension (DS Hi- Metal) contains colloidal silver nanowires with average length ~ 40 μm and diameter ~ 40 nm. We dilute it with IPA at a ratio 1:10 to obtain optimally thick electrodes. The film with the compliant electrodes are mounted on retaining frames. Wire bonding of flexible printed circuit board cables on the compliant electrodes finally yields a device unit ready for required test measurements.

### Static focal-tuning performance

**Figure 3:**
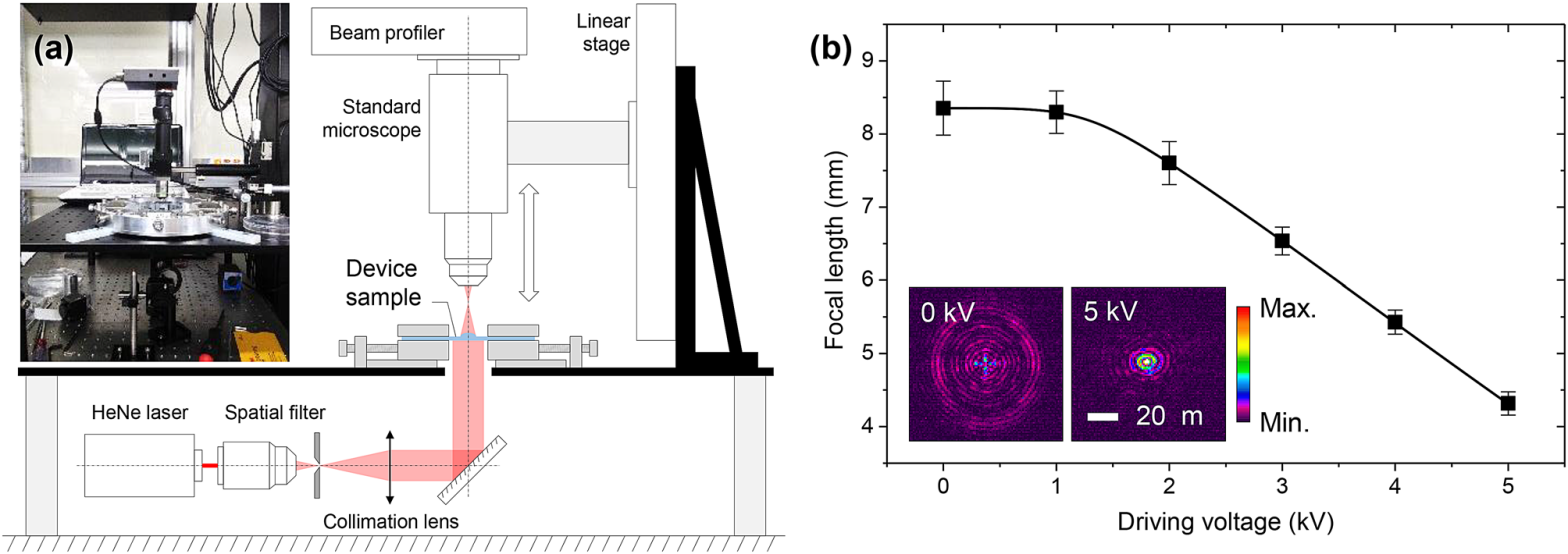
Focal-tuning range measurement. (**a**) Measurement set-up diagram and constructed system photograph. (**b**) Measured focal length *F*(*V*) for given driving voltage *V* changing over 0-to-5-kV range. Inset color-density plots show intensity distributions of the focal spots for 0 and 5 kV when the optical microscope is fixed at the on-focus condition for 5 kV. See Supplementary Video for a virtual image change due to this electrical focus-tuning operation.

We test fabricated device samples for their focal tuning ranges under electrical driving condition. In the focal tuning-range test, we use a modified optical microscope system indicated in Fig. 3a. In this system, the optical microscope consists of an objective lens (10×, WD 34 mm, NA 0.28), tube lens (focal length 200 mm, diameter 25.4 mm), and a beam-profiling image sensor (SP620U, Ophir Photonics). A carefully collimated HeNe-laser beam with effective diameter 12 mm and full-width divergence angle < 0.05 deg. is made incident on a device sample. A motorized linear stage (LTS300, Thorlabs) with 2- μm bi-directional precision is used to control location of the optical microscope along the optical axis.

In the measurement procedure, the optical microscope is tuned to an on-focus position *Z*_1_ for the device lens rim surface and then tuned to another on-focus position *Z*_2_ for the laser-beam focal spot formed by the device lens. Experimental focal length *F* is determined by *F* = *Z*_2_ − *Z*_1_. Empirical errors in the measured *F* in this scheme are less than 15 μm as estimated from tediously repeated trials on a fixed focal spot. This error is resulting dominantly from spherical-aberration-limited focal depth of the device lens.

Twenty independent device samples due to identical fabrication conditions optimized for off-state focal length at 8.3 mm are tested with this measurement scheme. The device samples in this measurement were fabricated with a metal mold with an engraved lens surface with diameter 0.77 mm, height 0.06 mm, and radius of curvature 1.18 mm that yields a resultant polymer lens with diameter 1.45 mm from the aforementioned process steps including a prestretch step by a radial strain 100%. The result is provided in Fig. 3b. Average focal length values are indicated by square symbols while vertical bars indicate ranges within the standard deviation. From this data, we confirm a reliable focal tuning range from *F* = 8.35 ± 0.37 mm at *V*_d_ = 0 to *F* = 4.32 ± 0.16 mm at *V*_d_ = 5 kV, which corresponds to 93.3% tunability in the relative quantity representation with respect to the minimal focal length in the tuning range. Inset color-density plots in Fig. 3b show intensity distributions of the focal spot at 0 and 5 kV when the optical microscope is on-focus condition for 5 kV, demonstrating how the focal spot changes on a fixed plane in response to the driving voltage variation. In Supplementary Video, we show variation of a virtual image due to this electrical tuning property within a 2-to-4 kV range.

Taking a closer look at the driving-voltage dependence of the average focal length, the curve shows a threshold-like trend around 1 kV and highly linear dependence from 2 to 5 kV, implying that the devices operate well below their elastic breakdown limits. The threshold-like behavior is related to an inactive response in the low driving-voltage region below 1 kV. This inactive region generally appears in DEAs using silver-nanowire random networks for compliant electrodes. This is considered to be a transient range where each nanowire yields only localized linear pressure points on the surface before the wire networks exert collective areal pressure over the entire electrode region, contributing to the in-plane actuation drive. Beyond 5 kV, the devices go into electrical discharge regions from around 5.2 ~ 5.4 kV which is an upper practical limit in our cases.

Importantly, the obtained focal-tuning response is induced by pure in-plane radial actuation and consequent change in the lens surface curvature, not by out-of-plane shift of the lens along the optical axis. Out-of-plane shift also enables an alternative focal-tuning mechanism in a similar DEA structure by producing a buckling deformation effect. However, the buckling deformation effect in DEAs is obtainable only for weak or no prestretching conditions with a strain value necessarily smaller than 10%^15,16^. Confirming the pure in-plane actuation of our device, we measure out-of-plane movement of our device with a laser scanning vibrometer (PSV-500, Polytec GmbH). Under driving voltage modulation in 0-to-4-kV range at 1 Hz modulation frequency, the out-of-plane displacement of the actuator membrane at the lens-rim surface is below 3 μm, which is only 7.5 × 10^−4^ of the obtained focal tuning range around 4 mm.

**Figure 4:**
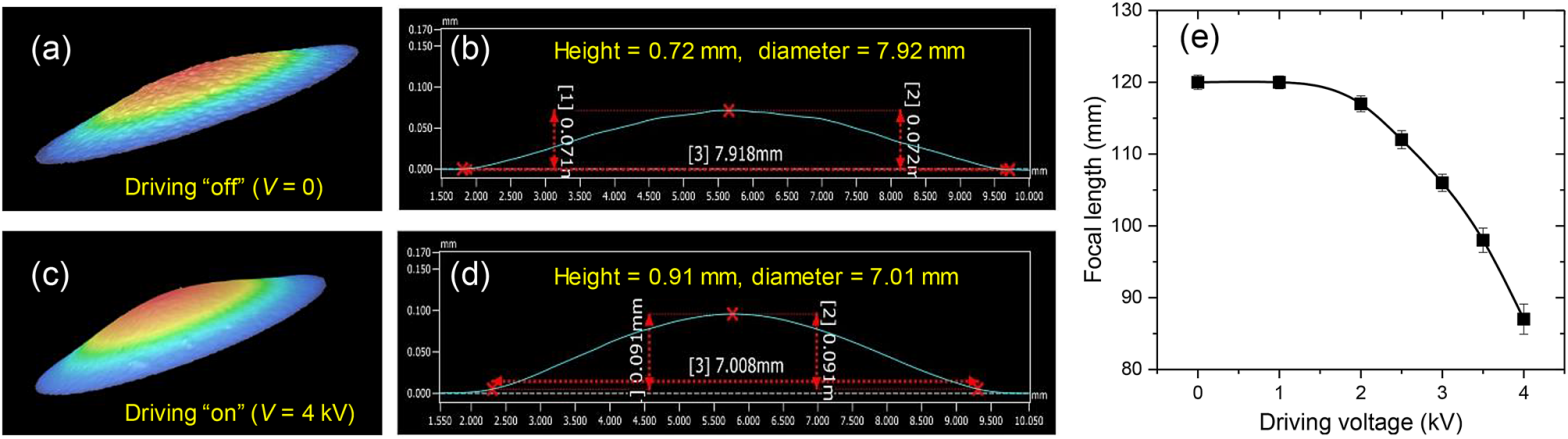
Electro-active radial actuation and focal-tuning range of a monolithic tunable lens with a longer focal length. (**a**) Three-dimensional surface topology and (**b**) cross-sectional surface profile of a tunable lens with a focal length 120 mm for driving voltage off (*V* = 0). (**c**) Three-dimensional surface topology and (**d**) cross-sectional surface profile of the same tunable lens for driving voltage on (*V* = 4 kV). This lens for a longer focal length is fabricated with a metal mold having an engraved hemi-spherical surface with diameter 4.17 mm, height 0.15 mm, and radius of curvature 14.5 mm. Under a static electrical driving condition, height/diameter of the lens surface topology change from 0.07 mm/7.92 mm at *V* = 0 to 0.09 mm/7.01 mm at *V* = 4 kV due to volume-preserving radial actuation. These surface profiles are measured with a commercial interferometric surface profilometer (VR-3000 3D Measuring Microscope, Keyence) (**e**) Measured focal-tuning range of this lens. Focal tuning range is 33 mm for driving voltage change from 0 to 4 kV.

In addition, we fabricate and test a tunable lens with a longer focal length ~ 120 mm in order to confirm lens surface-curvature change with the radial actuation and also to demonstrate its feasibility in commercial CCD or CMOS camera applications, as shown in Fig. 4. In Fig. 4a–d, the surface profiles measured with an interferometric laser surface profilometer clearly show lens surface-curvature change under the electrical driving conditions at *V* = 0 and 4 kV. From the measured surface profiles, we find no astigmatic profile difference between two orthogonal axes. Focal-length tuning operation in response to this surface-curvature change is provided in Fig. 4e. As the lens in this case has different diameter, height, and radius of curvature from the device for Fig. 3b, the measured driving-voltage dependence and focal tuning range are correspondingly different from the focal-tuning characteristics in Fig. 3b. Importantly, the obtained focal tuning range of 33 mm are presumably capable of imaging objects from 32 cm to the infinity from the lens surface without any mechanically moving parts, when estimated by the canonical thin-lens equation.

### Dynamic focal-tuning performance

Since our devices basically operate by physical movement of elastic body, high-speed response must be subject to a certain inertial speed limit. Therefore, we measure dependence of the focal-tuning range on frequency of a periodically modulated driving voltage signal. Dynamic focal-tuning range is indirectly measured by a confocal photodetector that replaces the beam-profiling image sensor in the measurement system indicated in Fig. 3a. The confocal photodetector consists of a pinhole with a diameter 10 μm and a Si-photodiode power sensor. A partial power of the laser beam that passes through the pinhole is measured by the confocal photodetector. Received signal by the confocal detector is maximal if an image of the focal spot is exactly on the pinhole. When the focal spot moves away from the maximum-signal position, the confocal photodetector signal decreases in proportion to the distance of the focal-spot movement. Therefore, signal received from the confocal photodetector maps the amount of focal shift of the device lens.

There are two reasons for using the indirect measurement here. First, it is technically difficult to construct an optical microscope that changes its working distance at a rate up to 300 Hz which is an upper bound of a frequency range of our interest. Second, response time of the beam-profiling camera is limited by a normal frame rate in the order of 10 frames per second. Therefore, the beam-profiling camera causes false analysis due to the rolling shutter effect. In contrast, response time of the confocal photodetector in our test is only 2 μs, enabling reliable measurement up to 250 kHz for periodic signals.

**Figure 5:**
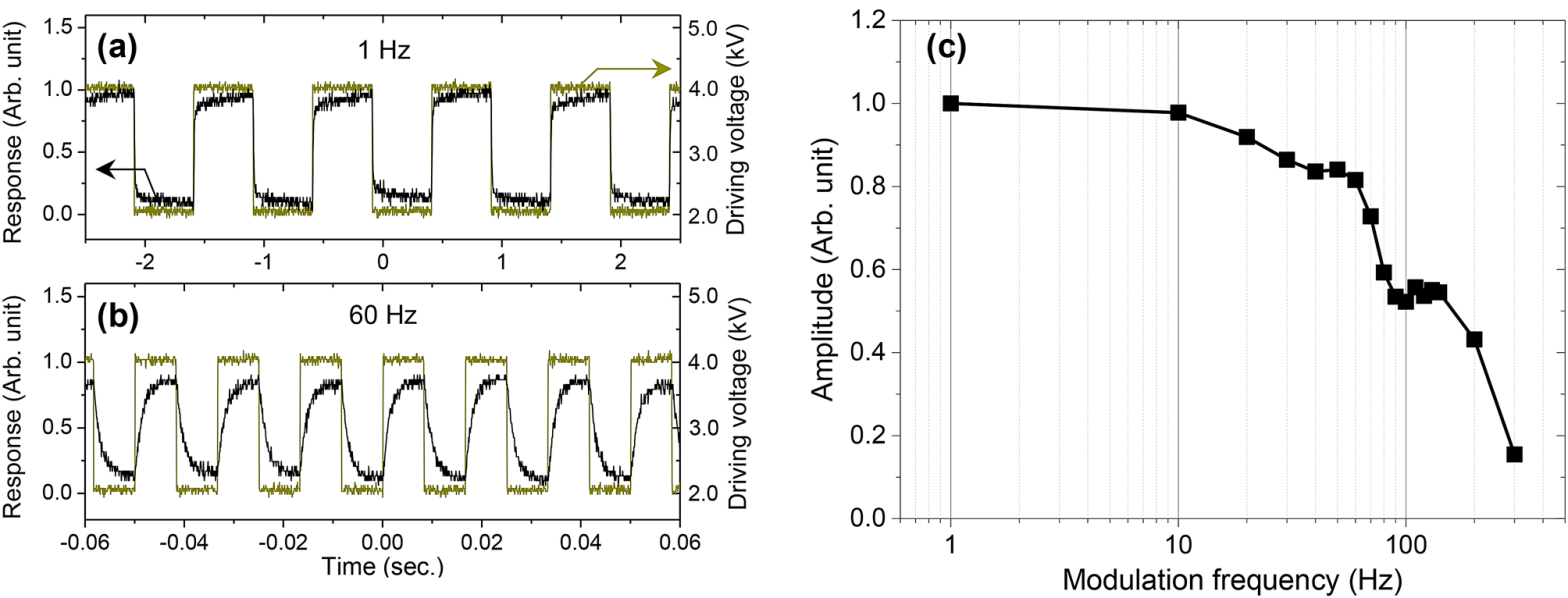
Focal-tuning speed measurement. (**a**) Focal-tuning response at modulation frequency 1 Hz. (**b**) Focal-tuning response at modulation frequency 60 Hz. Driving voltage signals are square waves alternating between 2 and 4 kV as indicated by dark-yellow profiles. (**c**) Relative focal-tuning range (modulation amplitude) as a function of modulation frequency.

In the measurement, we apply square-wave driving-voltage signal with a fixed duty cycle at 50% and modulation profile alternating from 2 to 4 kV, where the device samples linearly respond in the static operation conditions. The measurement result for a representative device sample is provided in Fig. 5. In Fig. 5a,b, response profiles received at the confocal photodetector for 1 and 60 Hz are indicated in comparison with the applied driving-voltage signals. At 1 Hz, the response profile shows almost instantaneous reaction with respect to the driving-voltage signal when the driving voltage jumps from 2 to 4 kV or vice versa. At 60 Hz, in contrast, modulation amplitude of the response profile reduces down to 82% of the 1-Hz modulation amplitude and the jump from one state to the other clearly shows normal exponential relaxation as the frequency approaches the inertial speed limit.

In order to determine the speed limit, we measure the modulation amplitude of the response profile as a function of driving-voltage signal frequency and the result is provided in Fig. 5c. From this data, we confirm that the upper cut-off frequency for a focal tunability of 50% with respect to the static tunability is ~ 140 Hz and corresponding dynamic response time is 7.1 ms. Importantly, this confirmed response time value is remarkably small when compared with ~ 67 ms for polylithic DEA tunable lenses in the previous study^13^. In further details from the dynamic response data in Fig. 5c, a wavy feature is found in 40 ~ 130 Hz region. This is caused by a low-Q resonant oscillation of the device film, which is inherent in stretched elastic polymer in general.

## Discussion

In conclusion, we have developed a monolithic focus-tunable lens based on the DEA technology. We established device fabrication processes including an optimal elastomer-film synthesis for desired actuation strength and elasticity. The fabricated devices show 93% focal tunability and 7 ms response time, which are remarkably enhanced performance characteristics from the previous polylithic DEA tunable lens devices. In particular, the high-speed characteristic is a unique feature of our monolithic DEA tunable lens technology that minimizes inertial resistance against desired elastic motion. For further development in the future, it is of great interest to apply our monolithic DEA tunable lens concept to emerging application areas such as robotics, drones, autonomous vehicles, and medical instruments.

## Supplementary information

Supplementary Information 1.

Supplementary Information 2.

## Data Availability

The data that support the findings of this study are available from the corresponding author upon reasonable request.
