# Supplementary material for: Monolithic focus-tunable lens technology enabled by disk-type dielectric-elastomer actuators
Source: Sci Rep. 2020 Oct 9;10:16937. doi: 10.1038/s41598-020-73666-0 (PMC7547700; doi:10.1038/s41598-020-73666-0)
Supplement: Supplementary file 2 — Supplementary Information 2. [file 41598_2020_73666_MOESM2_ESM.pdf]

Supplementary Figure for

# Monolithic focus-tunable lens technology enabled by disk-type dielectric-elastomer actuators

**Bong Je Park,[1] Suntak Park,[1] Meejeong Choi,[1] Seung Koo Park,[1] Sungryul Yun, Eunjin Shin,[1] & Jae Woong Yoon[2,*]**

[1]Artifical Intelligence Research Laboratory, Electronics and Telecommunications Research Institute (ETRI), Daejeon 34129, Korea

[2]Department of Physics, Hanyang University, Seoul 04763, Korea

[*]Corresponding author: jwyoon@hanyang.ac.kr

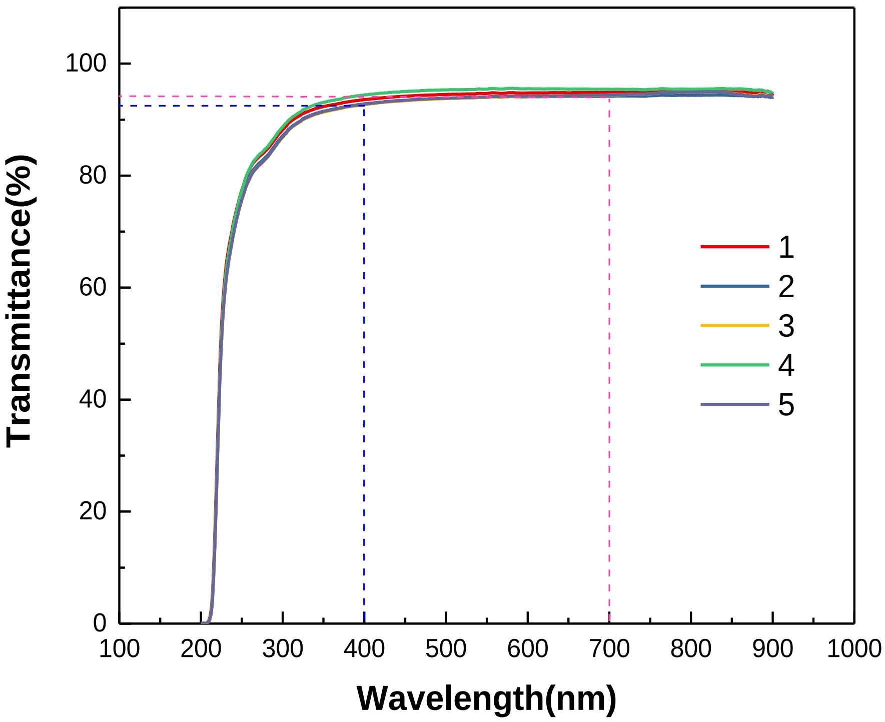


**Supplementary Figure 1. Optical transmittance spectrum of the optimized electro-active polymer film.** Five difference polymer-film specimens due to the identical optimized process conditions are measured with a commercial incoherent spectrometer (UV-2600 UV-VIS Spectrometer, Shimadzu). Film thickness values are fixed at 500 μm for all specimens. Average transmittance values are 93.25% at wavelength 400 nm and 94.66% at wavelength 700 nm.
